# Personal

**Published:** 1896-08

**Authors:** 


### Personal.

Dr. L. S. McMurtry, of Louisville, Ky., and his daughter, Marie-Louise, were recently the guests of Dr. and Mrs. William Warren Potter, of Franklin street, Buffalo. Dr. McMurtry sailed for Europe on La Gascogne, July 25, 1896, and will attend the Periodic Congress of Gynecology and Obstetrics, at Geneva. He is the official delegate of the American Association of Obstetricians and Gynecologists to the congress, and also is an accredited representative of the government of the United States to the same. He will return immediately on adjournment of the congress, which is to be held the first week in September.

---

Dr. Joseph M. Mathews, of Louisville, was recently reëlected president of the Kentucky state board of health. Dr. J. N. McCormack, of Bowling Green, was again chosen secretary. These capable and efficient officers should be retained in their present positions as long as they will consent to hold them.

---

Dr. Daniel Lewis has been elected for the second time president of the New York state board of health. This is a fitting recognition of the ability with which Dr. Lewis has administered the affairs pertaining to his office, and it is to be hoped he will be retained in it for many years.

---

Dr. George Ben Johnston, of Richmond, Va., was elected a Fellow of the American Surgical Association at its recent meeting, held at Detroit, Mich. This is a distinguished honor worthily bestowed. The association is a famous one and Dr. Johnston is an eminent surgeon.

---

Dr. Albert L. Gihon, of New York, medical director, U. S. Navy, (commodore, retired,) was elected president of the Association of Military Surgeons of the United States at its recent annual meeting at Philadelphia. The next annual meeting is appointed to be held at Columbus, O.

---

Dr. William C. Krauss, of Buffalo, has been designated as local correspondent of the *Cleveland Journal of Medicine.* If our Cleveland contemporary should have occasion to print something from the pen of Dr. Krauss, it will be material of value to the profession.

---

Dr. Henry Gibbons, Jr., of San Francisco, was elected president of the Medical Society of the State of California at its recent annual meeting. We predict one of the most successful years of the society under the able administration of Dr. Gibbons.

Dr. James F. W. Ross, of Toronto, will deliver the address on obstetrics and gynecology at the annual meeting of the Canadian Medical Association, to be held at Montreal, August 26, 27 and 28, 1896.

Dr. Thomas Bagley, of Buffalo, who recently visited Europe, has returned and resumed the practice of his profession.

